# The complete mitochondrial genome of *Apatura laverna* (lepidoptera: Nymphalidae)

**DOI:** 10.1080/23802359.2021.1970645

**Published:** 2021-09-02

**Authors:** Ju-Ping Wang, Yun-Fei Peng, Ping Gao, Tian-Wen Cao

**Affiliations:** a Shanxi Insect Herbarium, College of Plant Protection, Shanxi Agricultural University, Taiyuan, China; b Key Laboratory of Economic and Applied Entomology of Liaoning Province, College of Plant Protection, Shenyang Agricultural University, Shenyang, China

**Keywords:** *Apatura laverna*, complete mitogenome, phylogeny

## Abstract

*Apatura laverna* is subordinate to *Apatura* Fabricius of Nymphalidae, and endemic to China. The complete mitochondrial genome of *A. laverna* was sequenced and analyzed in the study. The length of the complete mitogenome is 15,187 bp, including 37 genes and a control region. *COI* gene initiate with a CGA codon, the rest 12 genes start with typical ATN. Eleven of 13 PCGs have a complete stop codon TAN except for *COII* and *ND4* have a single T. The phylogenetic analyses support that *A. laverna* has a close relationship with the clade including *A. metis* and *A. ilia.*

*Apatura laverna* Leech, 1892 is a typical forestry pest and distributed in Beijing City, Hebie Province, Henan Province, Shanxi Province, Sichuan Province and Liaoning Province in China (Wu and Xu 2017). The species is subordinate to *Apatura* Fabricius of Nymphalidae. Its host plant is Salicaceae plant such as *Salix caprea* Linnaeus and *Populus cathayana* Rehd. In this study, the complete mitogenome of *A. laverna* was sequenced and analyzed, which would provide useful genetic information for improving the taxonomic system and phylogenetics of Nymphalidae and provided scientific evidence for the identification and classification of *A. laverna* more correctly.

The specimen was collected from Luliang City, Shanxi Province, China (36.9876 N, 111.1189 E) in June 2016. The specimen was deposited at the Shanxi Insect Herbarium, College of Plant Protection, Shanxi Agricultural University (Tianwen Cao, ctwen@126.com) under the Voucher number QD20160617. Total genomic DNA was extracted using the OMEGA Insect DNA Kit. The Illumina TruSeq library was constructed and sequenced using the Illumina NovaSeq 6000 platform. A total of 6 Gb clean data were gained and assembled by IDBA-UD (Peng et al. 2012). The mitochondrial genome sequences were identified by the Geneious 10.1.3 (http://www.geneious.com/). The 22 tRNA genes were confirmed using tRNAscan-SE version 2.0.2 (Lowe and Chan 2016). The sequence data were deposited in GenBank under accession no. MF444860.

The complete mitochondrial genome of *A. laverna* is 15,187 bp in length, comprising two rRNA genes, 13 protein-coding genes, 22 tRNA genes, and a control region. The gene order and orientation are similar to the other Nymphalidae insects (Zhang et al. 2012; Wang et al. 2013; Song et al. 2016). The overall AT content was 80.15%, while the AT content of the *rrnL*, *rrnS* and control region were 84.76%, 85.66%, and 89.63%, respectively. Twelve genes start with typical ATN, while *COI* starts with CGA. Eleven of 13 PCGs have a complete stop codon TAN except for *COII* and *ND4* have a single T. The 22 tRNAs were interspersed throughout the whole mitogenome and ranged from 61 to 74 nucleotides.

The phylogenetic relationships were reconstructed based on 22 species of Nymphalidae and the other four butterfly families by Bayesian analysis. The *Hyphantria cunea* and *Lymantria dispar* were selected as outgroups. The protein-coding genes were aligned with the Mega 7.0 software (Kumar et al. 2016) and the datasets were conducted with Markov Chain Monte Carlo algorithm under GTR + I + G model using MrBayes3.1.2 (Huelsenbeck and Ronquist 2001). The phylogenetic analyses highly support the monophyly of butterflies (PP =1.00). The phylogenetic relationship of 5 butterfly families showed: (Papilionidae＋Hesperiidae)＋(Nymphalidae＋(Pieridae＋Lycaenidae)). The result is consistent with a similar previous study (Wang et al. 2016). The newly sequenced species *A. laverna* is subordinate to the Nymphalidae clade. The phylogenetic analyses support that *A. laverna* has a close relationship with the clade including *A. metis* and *A. ilia* by BI analyses at 100% (Figure 1).

**Figure 1.**
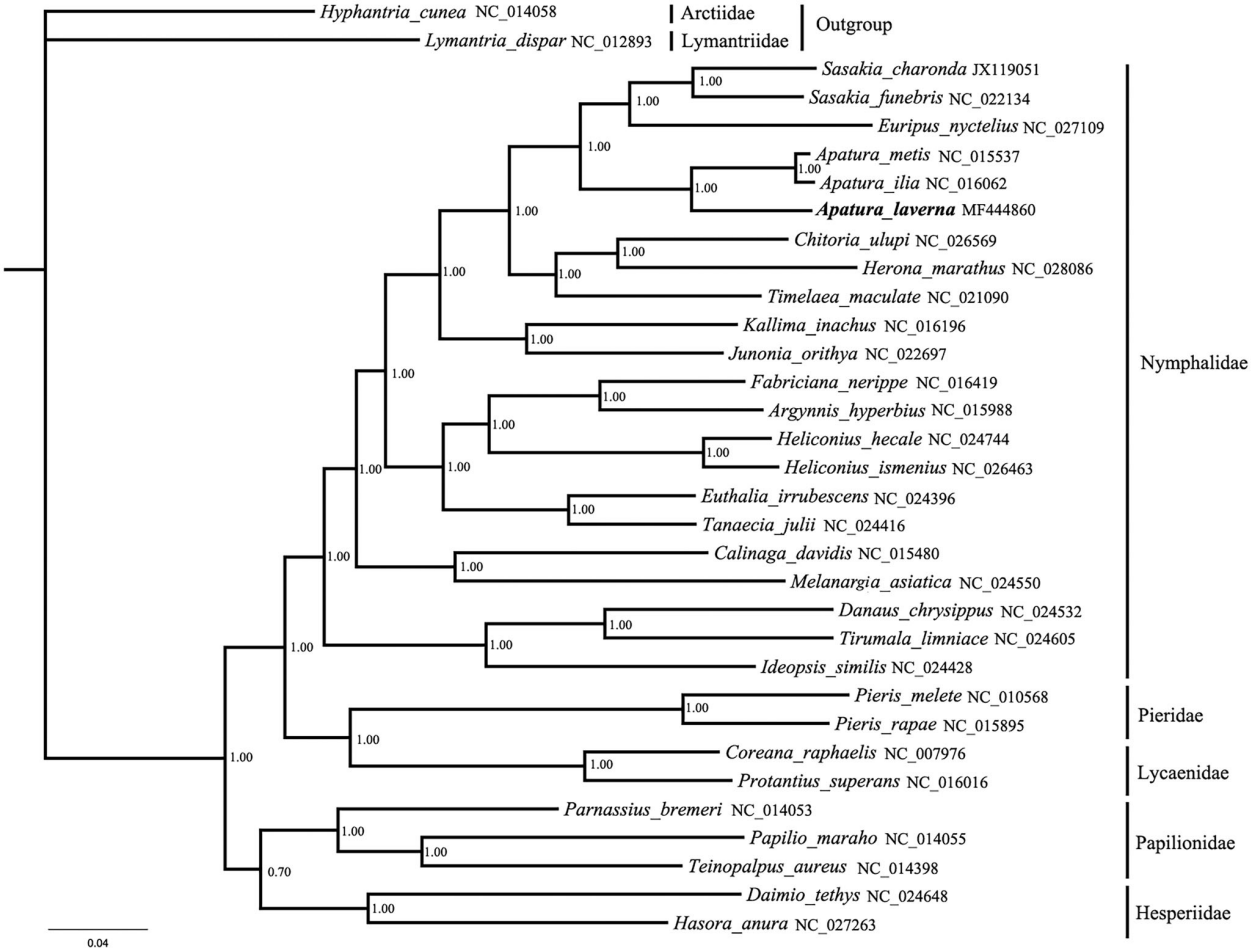
Bayesian phylogenetic tree based on 13 protein-coding genes of the mitochondrial genome sequences of 22 Nymphalidae species.

## Data Availability

The genome sequence data that support the findings of this study are openly available in GenBank of NCBI at https://www.ncbi.nlm.nih.gov under the accession no. MF444860. The associated BioProject, SRA, and Bio-Sample numbers are PRJNA732951, SRR14745268, and SAMN19355897, respectively.
